# An alternative norm of intention consistency

**DOI:** 10.1002/tht3.453

**Published:** 2020-04-06

**Authors:** Carlos Núñez

**Affiliations:** ^1^ Department of Philosophy University of Vienna Vienna Austria

**Keywords:** intention, intention consistency, means end coherence, practical rationality, rational requirements

## Abstract

In this paper, I formulate a norm of intention consistency that is immune to the kind of cases that have been put forth to argue either that rationality does not require consistency between an agent's intentions, or that, if it does, then rationality is not normative. The norm I formulate mimics refinements that have been made to the norm of means‐end coherence in response to cases where, intuitively, you need not be irrational when you intend an end *e*, despite not intending the means *m* you believe to be necessary for *e*, because you do not believe that *intending m* is necessary for *e*. Similarly, according to the norm I put forth, if you intend *e*, and believe that *e* is inconsistent with e*, you need not be irrational if you also intend e*, as long as you do not believe that intending e* is inconsistent with *e*.

## 1 SCEPTICISM ABOUT A NORM OF INTENTION CONSISTENCY

Many theorists of action believe that rationality requires of you that you do not intend each of two ends you believe to be inconsistent—in the sense that, were one of them to obtain, then because of that the other one wouldn't.^1^ Let me call this norm “End Consistency” (EC). According to it:

*End Consistency*. Rationality requires of an agent *A* that, if

1. *A* intends at time *t* that end *e*, and if
2. *A* believes at *t* that, were it to be the case that end e*, then because of that *not e*, then
3. *A* does not intend at *t* that e*.^2^^,^ 3

Some philosophers have resisted this idea. To do so, they point to cases where it seems like an agent would augment her chances of getting what she wants by intending each of two acknowledgedly inconsistent ends.

Consider a case that I take (and adapt) from Michael Bratman (1987):

**Video Game**: You are playing a video game in which you have to hit either one of two targets in order to win (call them ‘T1’ and ‘T2’). You have two guns and are ambidextrous enough to be able to aim and shoot at both. You know that your chances of hitting either target would be greater if you shot at both than if you shot only at one. You also know that the costs of shooting at both are the same as the costs of shooting only at one. However, you know that, were you to hit either one, then because of that the game would end and you would not hit the other.

Some people think that, in this situation, it isn't irrational for you to intend to hit T1 and intend to hit T2, despite your belief that these ends are inconsistent.^4^ This has led them to think that, either rationality does not require (believed) consistency between an agent's intentions, or, if it does, rationality is not normative.^5^

The reasons they have to think this are various. One reason is that, intuitively, it need not be irrational for you to try to hit T1 and try to hit T2. But trying to φ, they argue, necessarily implies intending to φ. So if it needn't be irrational for you to try to hit T1 and try to hit T2, it needn't be irrational for you to intend to hit T1 and intend to hit T2.^6^

Another reason they present is that, intuitively, it need not be irrational for you to adopt the goal of hitting T1 and also adopt the goal of hitting T2. But having the goal to φ, they argue, *just is* intending to φ. What it is to intend to φ, they would say, is simply to have φ‐ing as a settled object of pursuit. So if it needn't be irrational for you to be settled on the goal of hitting T1 and also be settled on the goal of hitting T2, it needn't be irrational for you to intend to hit T1 and intend to hit T2.^7^

One further reason could be that, intuitively, if you end up hitting T1, this is something you would have done intentionally and, moreover, rationally so. The same would go for T2. But intentionally φ‐ing, some argue, implies intending to φ.^8^ If one thinks this, then one may also think that, if it is rational for one to intentionally φ, then it must be rational for one to intend to φ. So, if hitting T1, or hitting T2, is in both cases something you would have done intentionally, and rationally so, if you do it at all, it must be that you rationally intended to hit T1 and and rationally intended to hit T2.

Perhaps these arguments can be resisted.^9^ But I shall not try to explore this issue here. Instead, I will simply formulate a norm of intention consistency that could be accepted by those who are convinced by such arguments, and so who think that, in cases like these, it need not be irrational to intend each of two acknowledgedly inconsistent ends.

More precisely, what I will do is revise the norm of consistency for intentions in ways that mimic revisions that have been made to the norm of means‐end coherence. I turn to this issue now.

## 2 LESSONS FROM MEANS‐END COHERENCE

Philosophers of action who think that rationality requires some kind of coherence between your intentions for ends and your intentions for means have generally come to accept that the means that should figure in a requirement of means‐end coherence are not those which you believe to be necessary for your ends, but those *the intending of which* you believe to be necessary for your ends.

To see this, consider John Broome's version of this requirement, which he calls the “Instrumental Requirement”:

*Instrumental Requirement*. Rationality requires of *A* that, if:

1. *A* intends at *t* that *e*, and if
2. *A* believes at *t* that, if *m* were not so, because of that *e* would not be so, and if
3. *A* believes at *t* that, if she herself were not then to intend *m*, because of that *m* would not be so, then
4. *A* intends at *t* that *m*.^10^

In Broome's terminology, a means *m* is “implied” by an end *e* iff, were *m* not so, then because of that *e* would not be so.^11^ Likewise, a means *m* is “up to” you iff, were you not to intend *m*, then because of that *m* would not be so. So the means that Broome's Instrumental Requirement concerns are only those you believe to be both implied by your end and up to you. In other words, only those *the intending of which* you believe to be implied by your end.

This restriction on the kind of means that figure in the requirement follows intuitions about cases where it seems like you need not be irrational even though you do not intend the means you believe to be implied by your end, because you do not believe you need to intend to take the means in order to take them. Broome presents the following example:

suppose you intend to fly to Venice tomorrow and believe your waking at six is a means implied by this end. But you know you are woken at six every morning by the braying of your neighbour's donkey. Then you do not need to intend to wake at six, because you believe that will happen anyway. (2013 p. 162)

As Broome notes, waking up at six it is not something you do. It is something that happens to you. So one could think that, if we restrict the means *m* in the requirement above to your actions, we could get rid of condition 3. But Broome, following Frances Kamm (2000), presents a case that shows that this isn't so:

You are a doctor and you intend to relieve the pain of one of your patients by giving her morphine. You believe that, in order to relieve her pain, you will have to give her so much morphine that you will kill her as a side‐effect… You do not [and need not] intend to kill her. You also intend to admit a new patient to your hospital. Since there are no spare beds, and you cannot move living patients out of the hospital, you believe you can admit a new patient only by killing an existing one. So you believe that killing a patient is a means implied by the end of admitting a new patient, which you intend. But that is all right, because you believe you will kill a patient anyway, as a side‐effect of your other intention to relieve her pain. You do not [and need not] intend to kill a patient. (2013 pp. 162–63)^12^

Killing a patient, unlike waking up, is something you do. Still, you may be rational despite not intending to do this, because you do not believe you need to intend to do it in order to do it.^13^

I think the overwhelming consensus is that there need not be any irrationality in cases like these (at least I know of no one who denies this). This has led theorists who think that rationality demands some form of means‐end coherence to reformulate the relevant requirement so that it concerns specifically those means *the intending of which* you believe to be implied by your end. Importantly, it hasn't led anyone to think that rationality does not require means‐end coherence, or that, if it does, rationality is not normative.

The main idea, then, seems to be the following: it need not be incoherent for you to intend *e* and not intend *m* when you believe that *m* is implied by *e*, because, although this belief relates *e* to *m*, it does not relate *e* to your *intending m*. That is, your belief leaves it open what the relation between *e* and your intending *m* is. Since it leaves this open, it could still be the case, according to your beliefs, that *e* even though you do not intend *m*. So this belief puts no structural pressure on you to intend *m*, despite your intention that *e*.

My point now will be simply that we could apply the same lesson to a requirement of intention consistency. I turn to this issue now.

## 3 END CONSISTENCY VS. END‐INTENTION CONSISTENCY

As far as I know, all cases that have been presented as intuition pumps against a norm of intention consistency are ones where, by pursuing each of two acknowledgedly inconsistent ends, an agent can augment her chances of getting what she ultimately wants.

One important feature of such cases is that, in them, you do not believe, of either one of the ends in question, that, were you to intend that end, then that end would come about. Because you do not believe this, you need not believe that, were you to intend one of the ends, then because of that the other would not obtain.

For instance, in Bratman's original case, you do not believe that, were you to intend to hit T1, you would hit it. You are not so confident of your own shooting abilities. The same goes for T2. This allows you to pursue both goals without ever coming to believe that you are doing something (or even holding any attitude) that will secure the frustration of your own pursuits. You can shoot at both targets and simply “let the world decide”—as Bratman puts it—which one, if any, you end up hitting.

This is a crucial feature of these cases. It is crucial that, in them, you can, by your own lights, pursue both ends in parallel without, so to say, stepping on your own toes, because at no point you believe, of anything you are doing (or of any attitude you hold), that it will secure the frustration of your own goals. This is precisely what allows theorists who appeal to such cases to claim that there is nothing going awry in these situations.

For, suppose instead that, besides believing that, were you to hit T1, you would not hit T2, you also believe that, were you to intend to hit T1, you would hit it. Perhaps you believe T1 is a pretty easy target. Since you are confident that, were you to intend to hit it, you would shoot at it, and that, were you to shoot at it, you would hit it, you believe you would hit it if you intended to do so. Because of this, you also believe (we can assume) that you would not hit T2 were you to intend to hit T1. In this scenario, it seems plausible that it *would* be irrational for you to intend to hit T1 and intend to hit T2. In deciding to hit T1 (by forming the intention to hit it), you would thereby be deciding not to do something you intend to do—namely, to hit T2.^14^ You would be, by your own lights, sabotaging your own pursuits.

Just as before with respect to the norm of means‐end coherence, then, it seems that what allows theorists to think that there need not be anything incoherent in these cases is that, although your belief relates *e* to e*, it does not relate *e* to intending e*. Your belief leaves it open what the relation here is. Since it leaves it open, it could still be the case, according to your beliefs, that *e* even though you intend e*. This is what allows such theorists to think that your belief puts no structural pressure on you to not intend e*, despite your intention that *e*.

If this is so, then there is an easy fix to the norm of intention consistency that would avoid such (putative) counterexamples. Let me call this the requirement of “End‐Intention Consistency” (EIC), to contrast it with the norm I called “End Consistency” (EC) before. According to it:

*End‐Intention Consistency*. Rationality requires of *A* that, if

1. *A* intends at *t* that *e*, and if
2. *A* believes at *t* that, were she herself then to intend that e*, then because of that *not e*, then
3. *A* does not intend at *t* that e*.

People who accept EC will most likely accept EIC. In any case, I see no motivation *whatsoever* to accept the former but reject the latter.

However, people who accept EIC need not accept EC. Anyone who is convinced that there is nothing rationally problematic in Bratman's case when you both intend to hit T1 and intend to hit T2, will reject EC. But they need not reject EIC.

Moreover, as far as I know, no one has presented a case where there is *any* intuitive pressure to reject EIC. So even people who have explicitly rejected a norm like EC could accept a norm like EIC.

In fact, whereas I see no reason whatsoever to reject EIC when you accept EC, I do think there is a genuine reason to accept the EIC even if you reject EC. The reason is that, if you only believe that *e* and e* are inconsistent, but you do not believe that *e* and intending e* are inconsistent, then, according to your beliefs, it could be the case both that *e* and that you intend e*. Because of this, one could think that there isn't, in this situation, structural pressure not to intend e*. One could think that you need the belief that *e* and intending e* are inconsistent to trigger such pressure.

## 4 CONCLUDING REMARKS

I have put forth a norm of intention consistency that is immune to the kind of cases that have been presented in the literature to reject the idea that rationality requires (believed) consistency between an agent's intentions (or that, if it does, it is not normative). I called this norm EIC, to contrast it with the more familiar EC.

I have not tried to argue that we should accept EIC. Nor have I argued that we should reject EC and accept EIC instead. For all I have said here, both, one, or neither one of these norms could be genuine norms of rationality. My only point here is that people who are convinced by the type of cases that have been presented so far to argue that EC is not a genuine requirement of rationality could still accept a requirement of EIC.

Now, some theorists who reject EC do not reject it simply because of such kind of cases. They reject it for the deeper reason that they deny that rationality requires any form of structural coherence as such (or because they think that, if it does, then rationality is not normative). They think that all that rationality (or normativity) requires is responding correctly to the reasons you have, or to the reasons that are somehow made available by your evidence. Following Bratman (2009), let me call them “myth theorists” about coherence requirements of rationality.^15^

It is interesting to note, then, that, since no case has been presented where there is any intuitive pull to the idea that you would have most reason to be end‐intention inconsistent,^16^ even myth theorists who deny that rationality requires any kind of formal coherence, and who deny, moreover, that, necessarily, if you are rational, you are end consistent, could still accept that, necessarily, if you are rational, you are end‐intention consistent.

Be this as it may, the fact that no case has been put forth that would present any intuitive challenge to EIC is a good reason to give it serious consideration in our theory of practical rationality.

## ENDNOTES

1 Among them, for example, Bratman (1987), Harman (1976), and Velleman (1989). They do not state the norm exactly as I do, but I think they would not oppose this formulation.

2 Where *e* and *e** are propositional letters.

3 A caveat: throughout this paper, and simply for ease of exposition, I will follow the customary practice of formulating these requirements and talking about them in terms of all‐out belief. I think doing so is problematic because the rationality of an agent's intentions shouldn't always be assessed relative to her beliefs. It should be assessed relative to the space of possibilities that she regards as open in deliberation, and belief does not always carve up that space. (On this point, see Núñez (2019)). Because of this, I think we should formulate these requirements in terms of that attitude, whatever we want to call it, that you hold towards a proposition whenever you deliberate conditional on it. Following Bratman (1992), we could call such an attitude “acceptance in a context.” Since it would be cumbersome for present purposes to switch to this manner of speaking, I will continue talking in terms of all‐out belief, and assume, simply for convenience, what I actually take to be false; namely, that, necessarily, if you believe that *p*, you deliberate conditional on *p*.

4 Interestingly, Bratman himself, who first popularized this type of case, does not think this. See Bratman (1987 ch 8).

5 For example, McCann (1986, 1991), who seems to hold the former view, and Kolodny (2008) who seems to hold the latter.

6 McCann (1986, 1991), Ludwig (1992), Adams (1986, 1995, 1997), and Kolodny (2008) think that trying to φ implies intending to φ. In part because they think this, McCann and Kolodny reject a norm of end consistency. Ludwig and Adams, however, do not.

7 For example, McCann (1986, 1991), and Kolodny (2008).

8 This is known as “the simple view” concerning the relation between intention and intentional action. It is defended, among others, by McCann (1986, 1991), Adams (1986, 1997), and Ludwig (1992). It is rejected, among others, by Bratman (1987), Harman (1976), and Mele (1992).

9 I myself happen to think that they *can* be resisted.

10 Broome (2013 p. 159). I changed Broome's ‘*N*’ for ‘*A’*. *m* is a propositional letter.

11 Broome formulates the requirement in terms of implied rather than necessary means. I think he is right in doing so, and I will here follow suit. Intuitively, you need not believe that intending *m* is strictly speaking necessary for *e* in order to be under rational pressure to intend *m* (or drop the intention for *e*). It would be enough to believe that, were you not to intend *m*, then because of that *e* would not obtain.

12 I present Broome's case, rather than Kamm's, because I find it somewhat simpler.

13 Kieran Setiya also convincingly argues for this idea in his (2007).

14 As I said in footnote 3, I am assuming for simplicity that, if you believe that *p*, you deliberate conditional on *p*. If we dropped this assumption, things would be different. For, suppose that, although you do believe that, were you to intend to hit T1, you would hit it, you are not certain of this. And suppose that, because you are not certain of this, you do not deliberate conditional on this belief. Instead, you deliberate on the basis of the probabilities you assign to the relevant contingencies. Since you think there is a slight chance that you do not hit T1 despite intending to hit it, it might make most rational sense for you to intend to hit each target, if the costs are negligible and doing so augments your chances of hitting either one. That being said, I shall ignore this complication here, and continue under the assumption that, if you believe *p*, you deliberate conditional on *p*.

15 Among them, the already mentioned McCann and Kolodny, and also theorists like Raz (2005), Kiesewetter (2017), or Lord (2018). As an anonymous referee points out, a different thesis that could also go under the “myth theory” label with respect to this issue is that so‐called “requirements” of coherence are really, when properly understood, *inviolable* descriptive principles that simply specify how attitudes like beliefs or intentions operate. (On this point, see for example, Finlay (2009) or Lee (2020)). I do not mean to refer to such theorists or such a thesis when I use the label.

16 At least barring appeals to state‐given reasons, which are irrelevant to the present dialectic because they do not present any problem that would be specific to EIC. To the extent that they can be used to challenge EIC, they can be used to challenge *any* requirement of coherence.
